# Contact X-ray Brachytherapy for Older or Inoperable Rectal Cancer Patients: Short-Term Oncological and Functional Follow-Up

**DOI:** 10.3390/cancers13246333

**Published:** 2021-12-16

**Authors:** Petra A. Custers, Barbara M. Geubels, Inge L. Huibregtse, Femke P. Peters, Ellen G. Engelhardt, Geerard L. Beets, Corrie A. M. Marijnen, Monique E. van Leerdam, Baukelien van Triest

**Affiliations:** 1 Department of Surgery, Netherlands Cancer Institute-Antoni van Leeuwenhoek, 1006 BE Amsterdam, The Netherlands; pa.custers@nki.nl (P.A.C.); b.geubels@nki.nl (B.M.G.); g.beets@nki.nl (G.L.B.); 2 Department of Radiation Oncology, Netherlands Cancer Institute-Antoni van Leeuwenhoek, 1006 BE Amsterdam, The Netherlands; f.peters@nki.nl (F.P.P.); c.marijnen@nki.nl (C.A.M.M.); 3 GROW School for Oncology and Developmental Biology, Maastricht University, 6200 MD Maastricht, The Netherlands; 4 Department of Surgery, Catharina Hospital, Postbox 1350, 5602 ZA Eindhoven, The Netherlands; 5 Department of Gastroenterology, Netherlands Cancer Institute-Antoni van Leeuwenhoek, 1006 BE Amsterdam, The Netherlands; i.huibregtse@nki.nl (I.L.H.); m.v.leerdam@nki.nl (M.E.v.L.); 6 Department of Radiation Oncology, Leiden University Medical Centre, 2300 RC Leiden, The Netherlands; 7 Division of Psychosocial Research and Epidemiology, Netherlands Cancer Institute-Antoni van Leeuwenhoek, 1006 BE Amsterdam, The Netherlands; e.engelhardt@nki.nl; 8 Department of Gastroenterology and Hepatology, Leiden University Medical Centre, 2300 RC Leiden, The Netherlands

**Keywords:** rectal cancer, contact X-ray brachytherapy, older patients, oncological outcome, functional outcome, quality of life

## Abstract

**Simple Summary:**

The cornerstone in rectal cancer treatment is total mesorectal excision, a major surgical procedure associated with morbidity and mortality, especially in older rectal cancer patients. To avoid major surgery, different radiotherapy techniques are being investigated. Studies on contact X-ray brachytherapy reveal promising oncological results. However, there are limited data on functional outcome and quality of life, which are highly important for older or inoperable patients. This study aims to report the oncological and functional outcome, quality of life, and patients’ experiences of older or inoperable rectal cancer patients treated with contact X-ray brachytherapy to avoid major surgery. This study shows that contact X-ray brachytherapy can provide a good tumor response and is well tolerated, with minimal impact on functional outcome and quality of life. These data suggest contact X-ray brachytherapy can be considered an option for older or inoperable rectal cancer patients to avoid major rectal surgery.

**Abstract:**

Total mesorectal excision for rectal cancer is a major operation associated with morbidity and mortality. For older or inoperable patients, alternatives are necessary. This prospective study evaluated the oncological and functional outcome and quality of life of older or inoperable rectal cancer patients treated with a contact X-ray brachytherapy boost to avoid major surgery. During follow-up, tumor response and toxicity on endoscopy were scored. Functional outcome and quality of life were assessed with self-administered questionnaires. Additionally, in-depth interviews regarding patients’ experiences were conducted. Nineteen patients were included with a median age of 80 years (range 72–91); nine patients achieved a clinical complete response and in another four local control of the tumor was established. The 12 month organ-preservation rate, progression-free survival, and overall survival were 88%, 78%, and 100%, respectively. A transient decrease in quality of life and bowel function was observed at 3 months, which was generally restored at 6 months. In-depth interviews revealed that patients’ experience was positive despite the side-effects shortly after treatment. In older or inoperable rectal cancer patients, contact X-ray brachytherapy can be considered an option to avoid total mesorectal excision. Contact X-ray brachytherapy is well-tolerated and can provide good tumor control.

## 1. Introduction

The mainstay in rectal cancer treatment is total mesorectal excision, in intermediate or locally advanced rectal cancer combined with neoadjuvant (chemo)radiotherapy [1]. Although this combined approach results in good oncological outcome, total mesorectal excision is associated with the risk of surgical morbidity and mortality [2]. Additionally, long-term bowel dysfunction is commonly observed after total mesorectal excision, especially when combined with neoadjuvant (chemo)radiotherapy, resulting in impaired quality of life of rectal cancer patients [3,4].

Older patients are more at risk for postoperative morbidity and mortality. Although the outcome of older rectal cancer patients following surgery has improved over the years [5,6], frailty is associated with increased postoperative complication rates, longer hospital stay, higher readmission rates, and decreased survival rates [7,8]. Compared to younger patients, the negative impact of postoperative complications on physical- and role functioning is significantly stronger in older patients [9]. Thereby, bowel dysfunction associated with total mesorectal excision can be troublesome for older patients and may result in loss of independence, affecting the quality of life.

Therefore, radiotherapy plays a key role in the treatment of older rectal cancer patients. Depending on level of frailty, limitations in clinical target volume (CTV) or a less toxic approach of 5 × 5 Gy instead of chemoradiation can be considered [10]. Additionally, a radiotherapy boost can be offered to aim for a clinical complete response and to avoid total mesorectal excision. Following a complete response, which is established in 16–27% of patients after neoadjuvant chemoradiotherapy (45–50.4 Gy) [11,12,13], a Watch-and-Wait approach can be considered. This non-operative approach consisting of strict surveillance as alternative for total mesorectal excision, tends to be an oncologically safe alternative for patients with a clinical complete response [11,14,15]. However, dose-response analyses estimate a radiotherapy dose of up to 92 Gy is necessary to establish a complete response in 50% of patients [16]. The limiting factor for such high radiotherapy doses is the potential toxicity of the normal tissue. To overcome this limitation, intracavity irradiation such as contact X-ray brachytherapy or high-dose-rate iridium brachytherapy can be offered. Combined with neoadjuvant (chemo)radiotherapy, both contact X-ray brachytherapy and high-dose-rate brachytherapy can provide significant downstaging and local tumor control [17,18,19,20,21]. Especially older or inoperable patients could highly benefit from intracavity irradiation to increase the proportion of patients in whom major surgery can be avoided.

Although functional outcome following high-dose-rate brachytherapy is reported in detail [22], most studies on contact X-ray brachytherapy provide little detail on functional outcome. Especially in older or inoperable patients, who are not necessarily treated with curative intent, the functional outcome and quality of life are highly important. Therefore, the aim of this study is to report oncological and functional outcome, quality of life, and patients’ experiences assessed with in-depth interviews of older or inoperable rectal cancer patients undergoing contact X-ray brachytherapy as a part of their treatment with the goal to avoid total mesorectal excision.

## 2. Materials and Methods

Since the introduction in December 2017, all consecutive patients treated with contact X-ray brachytherapy at the Netherlands Cancer Institute were included in a prospective single-center registry. This registry was approved by the local institutional review board, and informed consent was obtained from all patients.

For the current study, patients aged 75 years or older, or inoperable patients of any age with at least 6 month follow-up were selected from the prospective single-center registry. Patients were classified as inoperable by the multidisciplinary team because of a poor condition or severe comorbidity. All patients received additional contact X-ray brachytherapy for residual tumor following neoadjuvant therapy. Patients were eligible for contact X-ray brachytherapy when the residual tumor was within 12 cm of the anal verge and covered less than 2/3 of the rectal circumferential diameter. There were no strict criteria regarding neoadjuvant therapy; both patients treated with neoadjuvant (chemo)radiotherapy or local excision could receive additional contact X-ray brachytherapy. Patients were treated either to achieve a clinical complete response or to achieve local control of the residual tumor and the associated symptoms caused by the residual tumor. The latter group consisted of patients with residual tumor of 3 cm or more, with regrowth or progression of the residual tumor between neoadjuvant therapy and contact X-ray brachytherapy, or with evidence of distant metastases. For all patients, the Charlson Comorbidity Index was calculated before start of the treatment with contact X-ray brachytherapy [23].

### 2.1. Treatment

Before contact X-ray brachytherapy, the local tumor extent was evaluated with digital rectal examination (DRE), endoscopy, and MRI. Patients received contact X-ray brachytherapy using the Arianne Papillon 50 machine with a surface dose of 30 Gy per fraction using 50 kVp X-rays through the rectal treatment applicator. The procedure was performed in an outpatient setting for all patients. Enemas were given to clear the rectum. Patients were positioned in the lithotomy position. Before each contact X-ray brachytherapy application, the tumor was visualized by the radiation oncologist using rigid proctoscopy. Depending on the tumor size, the rectal treatment applicator was 20, 25, or 30 mm. Insufflation was used during positioning of the treatment applicator to visualize the tumor and to prevent irradiation of normal mucosal tissue. Patients received a total of three fractions with a two week interval resulting in a total dose of 90 Gy.

### 2.2. Follow-Up and Response Assessment

Tumor response evaluation and follow-up consisted of digital rectal examination (DRE) and endoscopy every three months during the first year and every six months thereafter. MRI for local response evaluation and CT for detection of distant metastases were not performed routinely but rather adapted to the individual patient and treatment goal. The endoscopic tumor response was scored by I.H. and P.C. at every follow-up moment using standard evaluation forms. The tumor response was categorized as 1. complete response (CR), defined as the absence of tumor featuring a white scare without any superficial ulceration or irregularity [24]; 2. partial response (PR), defined as ≥30% decrease of the tumor volume; 3. stable disease (SD), defined as <30% decrease or ≤20% increase of the tumor volume; and 4. progressive disease (PD), defined as >20% increase of the tumor volume [25]. The percentage of decrease or increase of the tumor volume was measured based on the maximum diameter and protrusion of the residual disease prior to treatment with contact X-ray brachytherapy.

### 2.3. Endoscopic Toxicity

Endoscopic toxicity was scored at the tumor site and the contralateral rectal wall (by I.H. and P.C.). A 3-point scale was used to evaluate the toxicity at the tumor site; 1. erythema/scarring, 2. superficial ulcer, and 3. deep ulcer without clear signs of residual tumor. The toxicity at the tumor site was scored at all endoscopies during follow-up, except for the endoscopies in which evident tumor was present.

Toxicity of the contralateral rectal wall was scored using the endoscopic proctitis assessment scale by Khan et al. [26]. The toxicity was scored as 0. normal mucosa; 1. mild erythema; 2. diffuse erythema and punctate hemorrhage; 3. frank hemorrhage; and 4. ulceration. The toxicity of the contralateral rectal wall was scored prior to contact X-ray brachytherapy and at every follow-up moment.

### 2.4. Assessment of Functional Outcomes and Quality of Life

Quality of life and functional outcome were assessed before treatment and at three, six, and twelve months after treatment with contact X-ray brachytherapy using self-administered questionnaires. Patients treated with total mesorectal excision for progressive disease did not receive questionnaires following surgery. Quality of life was assessed using the European Organization for Research and Treatment of Cancer Quality of life Questionnaires (EORTC-QLQ)-C30 and -CR29 (colorectal cancer specific quality of life). For this study, the following subscales or single-items were analyzed: Global health status, Physical functioning, Social functioning, Pain, Constipation, Diarrhea, Abdominal pain, Buttock pain, Bloating, Blood and mucus in stool, Flatulence, Fecal incontinence, Sore skin, and Stool frequency [27,28]. Over time, a difference of 10% in mean score was considered clinically significant [29].

To assess the functional outcome, the Low Anterior Resection Syndrome (LARS) score and the Vaizey incontinence score were used [30,31]. The LARS score differentiates between no (score 0–20), minor (score 21–29), and major LARS (score 30–42). The Vaizey incontinence score differentiates between minor (score 0–11) or major incontinence (score 12–24).

### 2.5. Patients Experience of Undergoing Contact X-ray Brachytherapy

Included patients were asked for an in-depth interview to evaluate the patients’ experience of undergoing contact X-ray brachytherapy and to improve the treatment workflow. The interviews were conducted by telephone, and a semi-structured questionnaire was used to guide the interview (see Supplementary Table S1). All interviews were recorded and transcribed verbatim. Two researchers, P.C. and B.G., independently analyzed the transcripts to develop a codebook containing the topics discussed in the interviews. Discrepancies were resolved in a consensus meeting. Finally, the topics were grouped into overarching themes for presentation purposes.

Approval from the local institutional review board was obtained to conduct in-depth interviews with the patients selected for the current study. All patients were asked to sign informed consent before the interview was conducted.

### 2.6. Statistical Analyses

Statistical analyses were performed using the Statistical Package for the Social Sciences (SPSS version 25.0, Inc., Chicago, IL, USA). The duration of follow-up was calculated from the date of the last fraction until the date of the last follow-up moment. The Kaplan-Meier method was used to estimate the 1. organ-preservation rate, defined as an in situ rectum; 2. progression-free survival, defined as the absence of local progressive disease; 3. and overall survival, defined as the absence of death. Patients were censored in case of loss to follow-up or death. Descriptive statistics were used to analyze the functional outcome, quality of life, and data of the in-depth interviews.

## 3. Results

Nineteen older or inoperable rectal cancer patients were prospectively included in the single-center registry, baseline characteristics are provided in Table 1. The median age was 80 years (range 72–91), most patients were male (68%), and had a Charlson Comorbidity Index of 4 or more (68%). The majority of patients were primarily diagnosed with a clinical staged cT3 tumor, and 32% had suspected locoregional lymph nodes. Fifteen patients (79%) were neoadjuvant treated with radiotherapy (chemoradiotherapy, 5 × 5 Gy, 13 × 3 Gy, or high-dose-rate brachytherapy), four patients (21%) with local excision. After a median interval of 3 months (range 1–30) following neoadjuvant therapy, patients were treated with contact X-ray brachytherapy. Eight patients (42%) were treated to achieve a clinical complete response, and eleven patients (57%) were treated to achieve local control of the residual tumor and the associated symptoms, such as control of rectal bleeding. Two patients received a total dose of 60 Gy instead of the standard 90 Gy. One of these patients was treated with local excision before contact X-ray brachytherapy and received chemoradiotherapy afterwards. The other patient was treated with high-dose-rate brachytherapy before contact X-ray brachytherapy.

### 3.1. Oncological Outcome

**Figure 1:**
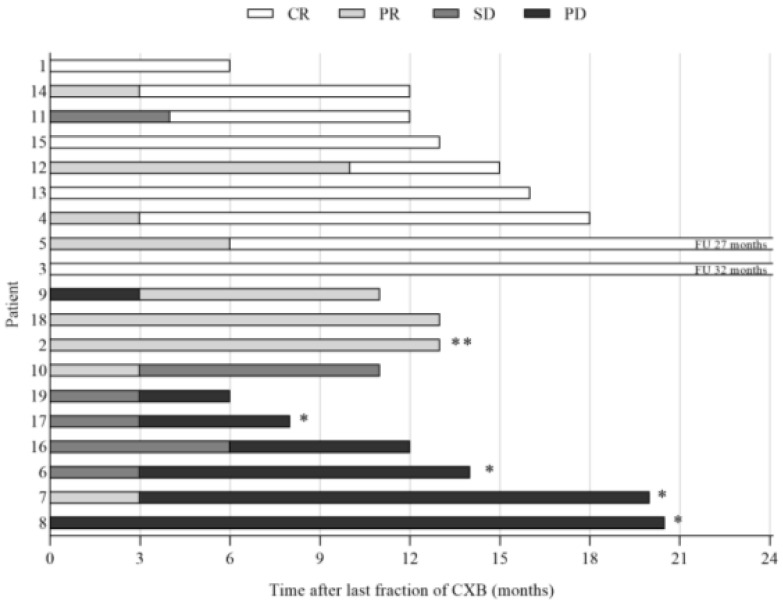
Response and survival. Abbreviations: CR = complete response; PR = partial response; SD = stable disease; PD = progressive disease; CXB = contact X-ray brachytherapy. * Four patients received salvage surgery. ** Deceased.

**Figure 2:**
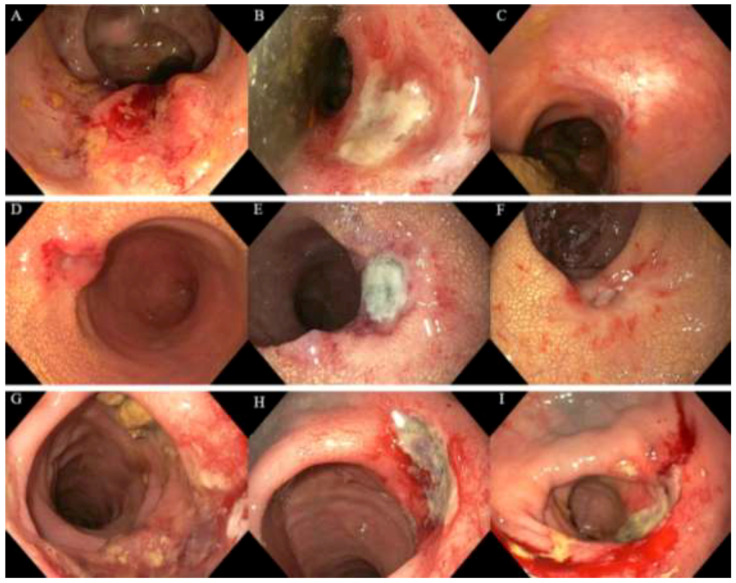
Endoscopic responses following contact X-ray brachytherapy of three patients. (**A**) Regrowth following chemoradiotherapy (50 Gy/25 fractions) for a cT3N0M0 rectal tumor before contact X-ray brachytherapy; (**B**) partial response showing a deep ulcer three months following contact X-ray brachytherapy (90 Gy/3 fractions); (**C**) endoscopic complete response six months following treatment; (**D**) residual tumor following radiotherapy (25 Gy/5 fractions) for a cT2N1M0 rectal tumor before contact X-ray brachytherapy; (**E**) partial response showing a deep ulcer three months following contact X-ray brachytherapy (90 Gy/3 fractions); (**F**) partial response showing a healing ulcer six months following treatment; (**G**) residual lesion of 4 cm following high-dose-rate brachytherapy for a cT2N0M0 rectal tumor before contact X-ray brachytherapy to achieve symptom control; (**H**) partial response showing a deep ulcer three months following contact X-ray brachytherapy (60 Gy/2 fractions); (**I**) sustained partial response showing a deep ulcer six months following treatment.

**Figure 3:**
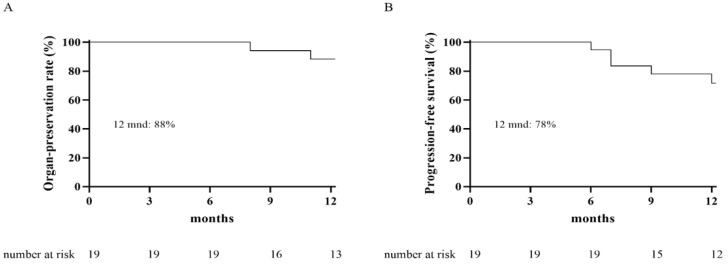
Kaplan-Meier curves for the organ-preservation rate (**A**) and progression-free survival (**B**).

After a median follow-up period of 13 months (range 6–32), local control of the tumor (CR, PR, SD) was observed in thirteen patients (68%), nine of whom had a clinical complete response. A partial response was observed in three patients, and stable disease was observed in one patient. Six patients (32%) had progressive disease of the tumor, four of whom subsequently underwent salvage surgery, best supportive care was given to two frail patients. The endoscopic tumor responses observed after contact X-ray brachytherapy are shown in Figure 1 and Figure 2. One patient was lost to follow-up after six months, and one patient died of other causes 13 months after treatment. The organ-preservation rate and progression-free survival at 12 months were 88% and 78%, respectively, see Figure 3. The overall survival at 12 months was 100%.

### 3.2. Toxicity Scored on Endoscopy

The endoscopic toxicity at the tumor site at three months was mild in 8/19 patients (erythema/scarring *n* = 4, superficial ulcer *n* = 4) and severe in 10/19 (deep ulcer). At six and twelve months, toxicity was mild in 10/19 and 10/13 and severe in 6/19 and 2/13 patients, respectively. The endoscopic toxicity at the tumor site was not scored when evident tumor was present, which was observed in 1 patient at three months, 3 patients at six months, and 1 patient at twelve months.

The endoscopic toxicity of the contralateral rectal wall was evaluable in seventeen patients; in two patients, the contralateral wall was not clearly visible. Prior to contact X-ray brachytherapy, the Khan score was 0 (normal mucosa) in one patient, 1 (mild erythema) in nine patients, and 2 (diffuse erythema and punctate hemorrhage) in seven patients. In none of the patients a score of 3 (frank hemorrhage) or 4 (ulceration) was seen. Following contact X-ray brachytherapy, the maximum Khan score was 0 in two patients, 1 in four patients, 2 in nine patients, and 3 in two patients. No ulcerations (Khan score 4) were seen.

### 3.3. Functional Outcome and Quality of Life

**Figure 4:**
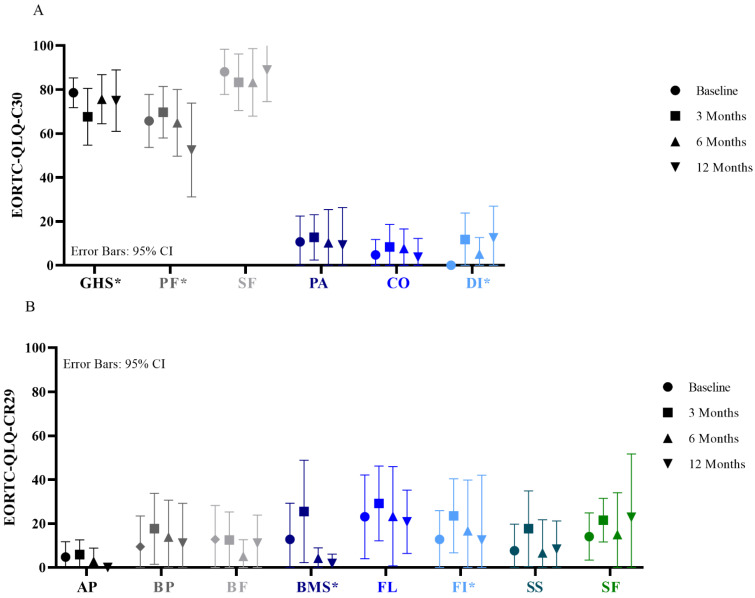
Quality of life according to the EORTC-QLQ-C30 (**A**) and EORTC-QLQ-CR29 (**B**). Functional scales, higher scores mean better results; GHS = Global health status; PF = Physical functioning; SF = Social functioning. Symptom scales, lower scores mean better results; PA = Pain; CO = Constipation; DI = Diarrhea; AP = Abdominal pain; BP = Buttock pain; BF = Bloating; BMS = Blood and mucus in stool; FL = Flatulence; FI = Fecal incontinence; SS = Sore skin; SF = Stool frequency. * Difference of 10 points is considered clinically relevant.

The EORTC-QLQ-C30 and -CR29 questionnaires were completed by 14/19 patients at baseline, 17/19 patients at 3 months, 13/19 patients at 6 months, and 9/14 patients at 12 months. At 3 months, a decrease of 10% in mean score in the subscale Global health status and an increase of 10% in the single-items Diarrhea and Fecal incontinence and subscale Blood and Mucus was observed compared to baseline. At 6 months, the subscales Global health status and Blood and Mucus, and the single-item Fecal incontinence improved back to baseline levels. Detailed results are shown in Figure 4.

**Figure 5:**
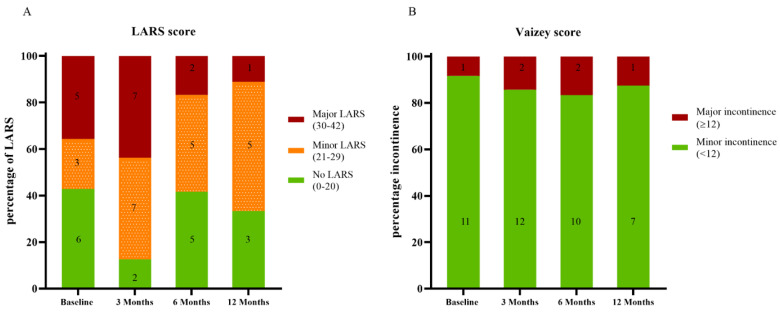
Defecation problems according to the LARS (**A**) and Vaizey score (**B**).

The LARS was completed by 14/19 patients at baseline, 16/19 patients at 3 months, 12/19 patients at 6 months, and 9/14 patients at 12 months. The Vaizey score was completed by 12/19 patients at baseline, 14/19 patients at 3 months, 12/19 patients at 6 months, and 8/14 patients at 12 months. Major LARS was observed in 5 (36%), 7 (44%), 2 (17%), and 1 (11%) patients at baseline, 3 months, 6 months, and 12 months after treatment. Major incontinence scored with the Vaizey score was observed in 1 (8%), 2 (14%), 2 (17%), and 1 (13%) patients at baseline, 3 months, 6 months, and 12 months after treatment. Detailed results are shown in Figure 5.

### 3.4. In-Depth Interviews

An in-depth interview was conducted with fourteen patients (74%) after a median of 16 months (range 1–34) following treatment. Two of these fourteen patients were treated with surgical resection for progressive disease resulting in a permanent colostomy. After eight interviews, saturation was reached; no new subthemes emerged. In the interviews, seven subthemes on the patients’ experiences of treatment with contact X-ray brachytherapy were identified, including the treatment itself, expectations of the treatment, treatment response, follow-up, side-effects, quality of life, and the overall patients’ reflection on the treatment, see Supplementary Table S2.

During treatment, pain was experienced by six patients, mainly during the placement or positioning of the applicator. Discomfort was experienced by eight patients, in four this was related to the lithotomy position. Side-effects during follow-up were described by ten patients, with blood loss as most common (*n* = 7). The majority of patients mentioned that the side-effects had no impact on their daily life. During the interviews, patients were asked to score their quality of life: 13 patients scored it as good. One patient reported poor quality of life, however, this was pre-existent and not worsened by treatment with contact X-ray brachytherapy. On reflection, all patients had a positive view regarding the treatment with contact X-ray brachytherapy and would choose again for this treatment or recommend treatment with contact X-ray brachytherapy to family and friends.

## 4. Discussion

This study reports on the oncological and functional outcome, quality of life, and experiences of older or inoperable rectal cancer patients treated with a contact X-ray brachytherapy boost to avoid total mesorectal excision. At the time of analysis, progression-free survival was seen in the majority of patients (68%); moreover, in almost half of the patients, a clinical complete response was achieved. Although a decrease in bowel function and quality of life was observed three months after treatment, these outcomes generally returned back to baseline levels at six months. Overall, patients’ experience with contact X-ray brachytherapy was positive.

Studies reporting on contact X-ray brachytherapy following external-beam radiotherapy show promising results. Ortholan et al. [32] reported on 88 patients (median age 67 and 69 years) treated in the randomized Lyon R96-02 study, and showed that a higher rate of clinical complete responses and organ-preservation can be established with an additional boost with contact X-ray brachytherapy. Sun Myint et al. [18] included 200 patients (median age 74 years) treated with contact X-ray brachytherapy and reported a clinical complete response rate of 72% and a 32-months organ-preservation rate of 62%. Dhadda et al. [17] treated 42 patients (median age 78 years) with T1-3 rectal cancer, resulting in a 2-year local regrowth-free, disease-free, and overall survival of 88%, 86%, and 88%, respectively. However, as the included patients were mainly treated with curative intent or had early-stage rectal tumors, they are not entirely comparable with the older or inoperable patients of this study; therefore, results are difficult to compare.

Studies on high-dose-rate brachytherapy did include older or inoperable rectal cancer patients. Rijkmans et al. [20] treated 38 patients (median age 83 years) with external-beam radiotherapy (13 × 3 Gy) followed by a three-weekly high-dose-rate brachytherapy boost (5–8 Gy). A clinical tumor response was observed in 29 patients (88%), 20 of whom had a clinical complete response. The 12 month local progression-free survival and overall survival were 64% and 82%. Garant et al. [21] treated 94 patients (median age 82 years) with external-beam radiotherapy (40 Gy in 16 fractions) followed by a three-weekly high-dose-rate brachytherapy boost (3 × 10 Gy). The clinical complete response rate and the 2-year local control rate was 86.2% and 71.5%. In both studies, patients were treated with high-dose-rate brachytherapy irrespectively of the tumor response after external-beam radiotherapy, whereas in the present study, only patients with residual tumor were treated; this might explain the difference in clinical complete response rate. However, all three studies show intracavity irradiation, such as contact X-ray brachytherapy or high-dose-rate iridium brachytherapy, can provide local tumor control in older or inoperable patients and can be an option to omit from total mesorectal excision.

In older or inoperable patients, functional outcome and quality of life might be more important than the oncological outcome. Therefore, in addition to the oncological outcome, this study provides detailed quality of life and functional outcome. Similar to other studies reporting on functional outcome after external-beam radiotherapy and intracavity irradiation, a decrease in bowel function and quality of life was observed in the first period after treatment [22,33]. The present study shows that this was generally restored to baseline levels at six months. Compared to the limited available literature on functional outcome after contact X-ray brachytherapy, the present study reported higher rates of major LARS. Dhadda et al. [17] reported no LARS in 65% of patients. Gérard et al. [19] reported the Memorial Sloan-Kettering anal sphincter function criteria (MSKCC score), a score which categories the sphincter function into four categories (excellent, good, fair, and poor) [34]. Of 64 patients, the sphincter function was good in 20 patients and excellent in 30. Additionally, of 25 patients, the LARS score was reported, major LARS was reported in one patient. Nevertheless, the patients in the current study, without total mesorectal excision, reported substantially less major LARS compared to patients treated with radiotherapy followed by total mesorectal excision, in which major LARS is reported in approximately 56% to 66% [4,35].

In addition to the self-administered questionnaires, in-depth interviews were conducted, revealing that all but one patient classified their quality of life after contact X-ray brachytherapy as good. Similar to previous study on dose-escalation with intracavity irradiation [17,19,36], in the current study, contact X-ray brachytherapy was given without compromising functional outcome and quality of life. Although side-effects were present in the majority of patients, these had no impact on their daily life. Moreover, all patients reflected positively on their treatment with contact X-ray brachytherapy. These results support the consensus statement made by the International Geriatric Radiotherapy Group, stating that radiotherapy with modern techniques is well tolerated by older rectal cancer patients [37].

The elegance of intracavity irradiation is the ability to give a high dose to the tumor and limited doses to the surrounding tissue [38]. However, at the tumor site, this high dose resulted in substantial toxicity in the first months after treatment, resulting in deep ulcers. These were observed in more than half of the patients at three months but were less common in the later period of follow-up. This might explain the observed transient increase in bowel dysfunction. The difference between high-dose-rate brachytherapy and contact X-ray brachytherapy is the amount of toxicity of the contralateral rectal wall, severe toxicity (deep ulcer) is not observed following contact X-ray brachytherapy, whereas in 7% of patients following high-dose-rate brachytherapy a deep ulcer at the contralateral wall is observed [22].

Radiotherapy plays an important role in the treatment of older or inoperable rectal cancer patients. The authors believe a boost with contact X-ray brachytherapy for older or inoperable rectal cancer patients with residual tumor following neoadjuvant treatment can be considered a valid alternative option to a total mesorectal excision. Although the authors encourage contact X-ray brachytherapy to be available for all older or inoperable patients, a boost should only be given in a dedicated center by a dedicated multidisciplinary team (radiation oncologist, surgeon, gastroenterologist, radiologist) to ensure the quality of the treatment and follow-up.

While this study reports in detail on functional outcome and quality of life, this study does have some limitations. First, this study reports on a small and heterogeneously patient group, therefore, the response rates and differences in functional outcome and quality of life have to be interpreted with caution. Second, the follow-up period was short, with a median of 13 months. Consequently, some patients will have progression or regrowth after this follow-up period; thus, a longer follow-up period is necessary to draw firm conclusions.

## 5. Conclusions

In older or inoperable rectal cancer patients, a radiotherapy boost with contact X-ray brachytherapy is well-tolerated and can provide a good tumor response with a local progression-free survival of 78% after 12 months. Even more important, the impact of contact X-ray brachytherapy on functional outcome and quality of life appears to be minimal, and patients experienced the treatment as positive. Therefore, contact X-ray brachytherapy can be considered an option for older or inoperable patients to avoid total mesorectal excision and the associated morbidity and mortality without compromising quality of life and functional outcome.

## Figures and Tables

**Table 1:** Baseline characteristics.

Total	*n* = 19	100%
Age, median (range), years	80	(72–91)
Gender		
Male	13	68
Female	6	32
Charlson Comorbidity Index		
2	3	16
3	3	16
≥4	13	68
Clinical T stage		
cT1	2	11
cT2	6	32
cT3	11	58
Clinical N stage		
cN0	13	68
cN1	5	26
cN2	1	5
Distance from anal verge		
0–5 cm	9	47
5–10 cm	9	47
10–15 cm	1	5
Differentiation		
Well	12	63
Moderate	1	5
Poor	0	0
Not known	6	32
Treatment prior to CXB		
Chemoradiotherapy ^1^	6	32
5 × 5 Gy	6	32
13 × 3 Gy	2	11
Local excision ^2^	4	21
HDR ^3^	1	5
Tumor size prior to CXB		
≤3 cm	16	84
>3 cm	3	16
Treatment intent		
Clinical complete response	8	42
Local control of the residual tumor	11	58
Dose of CXB		
90 Gy	17	89
60 Gy	2	11
Follow-up, median (range), months	13	(6–32)

Abbreviations: CXB, contact X-ray brachytherapy; HDR, high-dose-rate brachytherapy. ^1^ One patient received chemotherapy followed by chemoradiotherapy. ^2^ One patient received a dose of 60 Gy with CXB followed by chemoradiotherapy. ^3^ This patient received a dose of 60 Gy with CXB.

## Data Availability

Study data are available for review, upon reasonable request.

## Supplementary Materials

The following are available online at https://www.mdpi.com/article/10.3390/cancers13246333/s1, Table S1. Questionnaire in-depth interview; Table S2. Subthemes and quotes in-depth interview.
