# Supplementary material for: Contact X-ray Brachytherapy for Older or Inoperable Rectal Cancer Patients: Short-Term Oncological and Functional Follow-Up
Source: Cancers (Basel). 2021 Dec 16;13(24):6333. doi: 10.3390/cancers13246333 (PMC8699080; doi:10.3390/cancers13246333)
Supplement: Supplementary file 1 [file cancers-13-06333-s001.zip › cancers-1482040-supplementary.pdf]

Supplementary Materials

Table S1. Questionnaire in-depth interview.

**Experience of patients treated with contact x-ray brachytherapy**
Theme 1: Prior to treatment with contact x-ray brachytherapy<br>- How were you informed about the treatment? Did you miss any information about the treatment? Would you prefer to get informed in a different way?<br>- Did you get enough information about possible alternative treatments?<br>- Did you have enough time to make the medical decision with your physician?
Theme 2: Treatment with contact x-ray brachytherapy<br>- Was the intent of the treatment clearly explained to you?<br>- How did you experience the treatment?<br>- Was the treatment as you expected, based on the information you got prior to the treatment?<br>- How did you experience the personal care during the treatment?
Theme 3: The result after treatment with contact x-ray brachytherapy?<br>- What was the result you expected to achieve after the treatment with contact x-ray brachytherapy?<br>- How satisfied are you with the result?<br>- Did you experience any complaints or side-effects during or after treatment with contact x-ray brachytherapy? If yes, which complaints or side-effects?<br>- Where those complaints or side-effects discussed with you prior to the treatment?<br>- Where those complaints or side-effects as you expected?<br>- How do you experience your quality of life at this moment after treatment with contact x-ray brachytherapy?<br>- How is your quality of life compared to your quality of life before treatment with contact x-ray brachytherapy?<br>- Is the quality of life at this moment as you expected it to be, based on the discussion with your physician prior to treatment with contact x-ray brachytherapy?<br>- Can you handle the uncertainty or stress accompanied with the follow-up investigations well?
Theme 4: Conclusion<br>- If you have to evaluate everything, so the treatment itself, the first period after the treatment, possible side-effects, and the result of the treatment, did you all expect this?<br>- Would you choose a second time for the treatment with contact x-ray brachytherapy? Would you recommend the treatment to family or friends?<br>- On a scale of 1 to 10, how satisfied are you with the treatment?<br>- On a scale of 1 to 10, how satisfied are you with the result of the treatment?<br>- On a scale of 1 to 10, how satisfied are you with you quality of life at this moment?

Table S2. Subthemes and example quotes in-depth interview.

**Subtheme**	**N=14 (100%)**	**Example quotes**
Experiences with treatment		
Discomfort	8	"It is not pleasant, though not painful, it has to be done anyway" (patient 12)
Painful	6	"I did have terrible pain, (…), but as soon as the applicator was out of my body it was okay for me" (patient 3)
Expectations regarding treatment with contact x-ray brachytherapy		
Like expected	8	"The doctors established a realistic expectation pattern before start of the treatment" (patient 11)
Expected otherwise	3	"I did not expect it to be so painful" (patient 3)
Treatment response		
Satisfied	11	"I'm satisfied, I'm happy that it all went well" (patient 12)
Disappointed	2	Eventually, the expectations I had of the treatment were higher than what I got, but that's the way it is" (patient 6)
Follow-up		
Stressful	7	"Every follow-up moment, I certainly do experience some anxiety, but it is not a heavy burden for me" (patient 10)
Not stressful	6	"I don't think it is stressful at all, to be honest, I think it [the follow-up appointment] is quite interesting" (patient 11)
Side-effects		
Present	10	"I did experience some blood loss after the treatment" (patient 13)
Not present	4	"I did not experience any side-effects" (patient 16)
Quality of life		
Good quality of life	13	"My quality of life is fine, I don't experience any side-effects, but I never have" (patient 10)
Poor quality of life	1	"Well, I will not say it is 0.0, but my quality of life is not so good, especially due to my incontinence" (patient 3)
Patients' reflections on the treatment		
Positive	14	"I would do it again right away" (patient 9)
